# Higher Plasma Myostatin Levels in Cor Pulmonale Secondary to Chronic Obstructive Pulmonary Disease

**DOI:** 10.1371/journal.pone.0150838

**Published:** 2016-03-21

**Authors:** Chun-rong Ju, Miao Chen, Jian-heng Zhang, Zhi-ya Lin, Rong-chang Chen

**Affiliations:** State Key Lab of the Respiratory Disease, Guangzhou Institute of Respiratory Disease, First Affiliated Hospital of Guangzhou Medical University, Guangdong, China; Scuola Superiore Sant'Anna, ITALY

## Abstract

**Objective:**

To analyze plasma myostatin levels and investigate their relationship with right ventricular (RV) function in patients with cor pulmonale secondary to chronic obstructive pulmonary disease (COPD).

**Methods:**

The study recruited 81 patients with advanced COPD and 40 age-matched controls. The patients were divided into two groups: those with cor pulmonale and those without. Echocardiography was used to evaluate RV function and morphology, and the value of tricuspid annular plane systolic excursion (TAPSE) less than 16 mm was considered RV dysfunction. Plasma myostatin levels were analyzed by enzyme-linked immunosorbent assay, and B-type natriuretic peptide (BNP) levels were analyzed as a comparison of myostatin.

**Results:**

The data detected cor pulmonale in 39/81 patients, with the mean value of TAPSE of 14.3 mm. Plasma myostatin levels (ng/mL) were significantly higher in patients with cor pulmonale (16.68 ± 2.95) than in those without (13.56 ± 3.09), and much higher than in controls (8.79±2.79), with each *p*<0.01. Significant differences were also found in plasma BNP levels among the three groups (*p*<0.05). Multivariate regression analysis suggested that myostatin levels were significantly correlated with the values of TAPSE and RV myocardium performance index among the COPD patients, and that BNP levels were significantly correlated only with systolic pulmonary arterial pressure, with each *p*<0.05.

**Conclusions:**

Plasma myostatin levels are increased in COPD patients who have cor pulmonale. Stronger correlations of plasma myostatin levels with echocardiographic indexes of the right heart suggest that myostatin might be superior to BNP in the early diagnosis of cor pulmonale in COPD.

## Introduction

Cor pulmonale is a common heart disease, which is closely associated with chronic obstructive pulmonary disease (COPD) and the most common cardiac complication in the natural history of COPD [1]. Cor pulmonale has emerged, in recent years, as a leading cause of disability, and death with COPD is becoming increasingly prevalent [2]. The term “cor pulmonale” is generally used to describe the form of right heart dysfunction and hypertrophy. It is a progressive condition with initial compensatory right ventricular (RV) dysfunction and is becoming overwhelmed by increased RV systolic requirements, while left ventricular function remains relatively preserved [3].

RV dysfunction is common in patients with advanced COPD and more pronounced in the presence of pulmonary arterial hypertension (PAH) [4]. However, new research has shown that cardiac complications including RV dysfunction and hypertrophy start early in the course of the disease even at subclinical levels of PAH [5]. This means that PAH is not the sole pathological determinant of cor pulmonale in COPD. Actually, direct effects of smoking on RV dysfunction and remodeling have been demonstrated [6]. Also, it was reported that hypoxemia could result in RV hypertrophy directly [7]. Moreover, a study has shown that the survival of patients is not so much linked to the values of PAH; rather it is linked to the dysfunction of the right heart [8]. These results raise the awareness of the importance of early diagnosis of RV dysfunction, although it is difficult in patients with COPD, especially in those without prominent PAH.

Myostatin, growth differentiation factor 8, was initially found in several diseases involved in skeletal muscle wasting [9], [10]. Currently, myostatin is well recognized to play an important role in regulating muscle mass and function as a new member of transforming growth factor superfamily [11]. Recent studies found that myostatin levels were also elevated in the left ventricle of the patients with left heart failure [12], and that the elevated circulating myostatin levels were associated with left heart failure [13]. However, these studies mainly focused on the left heart. Very few data on the relationship between myostatin and cor pulmonale are available. A previous study demonstrated increased levels of circulating myostatin in patients with COPD; however, the RV function was not investigated in this population [14]. As COPD primarily affects the right side of the heart and leads to cor pulmonale, it was supposed that myostatin levels might be associated with RV dysfunction in cor pulmonale in COPD. Therefore, the present study aimed to determine the potential relationship between circulating myostatin concentrations and RV dysfunction in advanced COPD by measuring plasma myostatin levels in this population. The study also analyzed plasma levels of B-type natriuretic peptide (BNP) as a comparison of myostatin because BNP is established as a biomarker of RV dysfunction in chronic lung disease [15].

## Materials and Method

### Study Design

This study was a cross-sectional registry study conducted at a teaching hospital which was affiliated to Guangzhou Medical University. The study protocol was approved by the Ethics Committee of Guangzhou Medical University. Each participant provided a signed informed consent form before recruitment. The study recruited 81 patients with COPD and 40 controls. All subjects underwent a standard evaluation by means of medical history, routine clinical examination, spirometry measurement, transthoracic echocardiography, and plasma myostatin and BNP level analysis.

### Study Subjects

All patients were recruited prospectively in the outpatient department of the Guangzhou Institute of Respiratory Disease in the hospital between October 2012 and December 2014. The patients were kept under care in outpatient clinics specialized in respiratory medicine at the time of inclusion in the study. The inclusion criteria were as follows: (1) age > 50 years, (2) clinically and spirometrically confirmed COPD based on the GOLD Guidelines [16], (3) in GOLD stage III or IV, (4) clinically stable for at least 8 weeks at the time of enrollment, and (5) nonsmokers or ex-smokers who had abstained smoking for at least 3 years. The exclusion criteria were as follows: (1) the presence of left heart disease or left ventricular dysfunction (ejection fraction <50% on echocardiography) due to ischemic heart disease, cardiomyopathies, congenital heart diseases, valvular heart disease, or other cardiovascular disease; (2) malignancy, metabolic conditions, malnutrition, muscular disease, systemic inflammatory diseases, or renal failure; (3) cor pulmonale that originated from idiopathic pulmonary hypertension or other respiratory problems such as interstitial lung disease, sleep-disordered breathing, and tuberculosis; and (4) the patients who were on systemic corticosteroids at the time of enrolment or within 8 weeks of the study time. The patients were classified into two subgroups: COPD with cor pulmonale and without. Cor pulmonale was diagnosed by an experienced respirologist based on an established clinical history of cor pulmonale or the current clinical evidences, chest radiography, electrocardiogram, and echocardiography. The clinical signs included peripheral edema, jugular venous distension or tender hepatomegaly, increased intensity of pulmonary component of the second heart sound, and systolic murmur of tricuspid regurgitation [16], [17]. Chest radiography demonstrated an increased width of right descending pulmonary artery or RV enlargement. Electrocardiogram presented with P pulmonale, right axis deviation, or RV hypertrophy [3], [16]. With regard to echocardiography, RV hypertrophy or dilation accompanying RV dysfunction was required for the enrollment. The value of tricuspid annular plane systolic excursion (TAPSE) less than 16 mm was the sine qua non of RV dysfunction in the study, according to the recommendations [18], [19]. Thus, patients with TAPSE <16 mm were placed in the cor pulmonale group.

### Pulmonary Function Tests

All participants underwent spirometry and reversibility testing with the inhalation of a short-acting β_2_-agonist of salbutamol (400 μg). The main parameters of spirometry included forced expiratory volume in the first second (FEV_1_), forced vital capacity (FVC), ratio of FEV_1_/FVC, and FEV_1_ percentage of the predicted value (FEV_1_%pred) after bronchodilator administration.

### Transthoracic Doppler-Echocardiography

All study participants underwent Doppler-echocardiographic examination. The recordings were performed from left parasternal long- and short-axis and apical four-chamber views, adjusted to acquire the RV-focused view. Parameters of right heart morphology and function were measured based on the guidelines of European Respiratory Society of Echocardiography [20], [21]. The main morphology measurements included RV free wall thickness, RV end-diastolic diameter, and right atrial major diameter. As for the main functional parameters, TAPSE was measured and RV myocardial performance index (RVMPI) was derived. The RV fractional area change (FAC) was calculated based on the measurements of the RV end-diastolic and end-systolic areas [21].

Continuous wave Doppler was used to detect tricuspid regurgitation and calculate the systolic pulmonary artery pressure (SPAP). PAH was considered when the tricuspid regurgitation velocity was >2.8 m/s at rest, which was equivalent to the SPAP value above 36 mmHg, based on the guidelines [22], [23]. No major discrepancies were reported among echocardiogram operators in the performance of the echocardiography measurements.

### Analysis of Myostatin and BNP

For each subject, peripheral venous blood was drawn in the early morning after an overnight fasting. Plasma samples were deep frozen and kept at –80°C until assay after centrifugation at 1000 rpm for 5 min at 4°C. Total myostatin levels were measured using commercially available competitive antibody sandwich enzyme-linked immunosorbent assay (ELISA) kits (BioVendor Laboratories Ltd, Uscn Life Science Inc. China) based on polyclonal antibodies raised against recombinant human myostatin in rabbits [24]. Plasma BNP levels were analyzed by ELISA kits (Boster Biotch Co. Ltd., Wuhan, China). Both assays were performed according to the manufacturer’s specifications.

### Statistical Analysis

Statistical analysis was performed using SPSS 16.0 package (SPSS Inc, IL, USA) and GraphPad Prism v. 5 (GraphPad Software Inc., CA, USA). Continuous data were reported as means ± standard deviation. Categorical variables were summarized by proportion in each category and compared using chi-square test. Analysis of variance (ANOVA) tests were used to analyze differences among the three groups including controls and two subgroups of COPD (with cor pulmonale and without). Pearson's correlation analysis was performed with the coefficient (*r*) presented to investigate the relationship between plasma myostatin levels and other parameters in all of the patients. Multivariate regression models were then developed using the stepwise method to determine the selected parameters independently contributing to plasma myostatin levels. In the stepwise regression analysis, Alpha-to-Enter 0.15 and Alpha-to-Remove 0.15 were included. The correlation between plasma BNP levels and other parameters were also investigated using Pearson's correlation analysis. The level of statistical significance was set at *p*<0.05.

## Results

### Characteristics of Study Subjects

Eighty-one patients with COPD were compared with a group of 40 controls who were determined to be healthy based on clinical, spirometric, biochemical, and imaging investigations. Age was matched among the groups; however, there were significant differences in body mass index (BMI) between the patients and controls though they were recruited by cluster sampling. Among the patients with COPD, spirometry tests identified 29 (35.8%) patients in GOLD stage III and 52 (64.2%) in stage IV, with their mean FEV_1_ of 35.3% pred and 22.8% pred, respectively. All the patients recruited in this study were on inhaled corticosteroids + long-term bronchodilators (ICS+LABA), with some patients combined on tiotropium bromide. Chi-square test showed that there was no difference in the medication between the patients with or without cor pulmonale (Table 1).

**Table 1:** Clinical Data in Controls and Two Subgroups of COPD (Cor Pulmonale and No Cor Pulmonale) (Mean±SD).

Variables	Controls (n = 40)	No cor pulmonale (n = 42)	cor pulmonale (n = 39)	F value	*P* value
Age (years)	64.75±6.03	64.36±6.10	66.67±6.93	1.51	0.23
Sex, male (%)	21 (53%)	36 (86%)^†^	34 (87%)^†^	15.86	<0.01
BMI (kg/m^2^)	22.75±1.37	19.09±2.74^††^	18.61±2.99^††^	33.77	<0.001
FEV_1_ (L)	2.66±0.54	0.77±0.20^††^	0.55±0.19^††^*	435.90	<0.001
FEV_1_%pred (%)	97.99±7.97	30.41 ±7.42^††^	22.84±7.58^††^**	1159.84	<0.001
FEV_1_/FVC (%)	90.13±12.43	39.34±5.95^††^	31.28±6.71^††^**	436.38	<0.001
SBP (mmHg)	125.6±11.08	133.98±7.37^††^	133.82±8.25^††^	11.34	<0.001
DBP (mmHg)	74.55±5.64	80.17±5.73^††^	80.46±6.49^††^	12.54	<0.001
SPO_2_ (%)	97.81±1.96	91.74 ±1.59^††^	89.54±2.29^††^**	190.79	<0.001
Smoking (pack*years)	3.15±7.52	28.24±18.22^††^	26.48±18.88^†^	31.74	<0.001
No-/Ex-smoker	26/14	7/37^††^	6/31^††^	28.97	<0.001
Gold stage III/ IV	-	22/20^††^	7/32^††^	8.99	0.003
ICS+LABA/ LAMA	-	25/17	23/16	2.78	0.10

BMI-body mass index, COPD-chronic obstructive pulmonary disease, DBP-diastolic blood pressure, FAC-fractional area change, FEV1-forced expiratory volume in first second, %pred-percent of predicted value, FVC-forced vital capacity, Gold-global initiative of obstructive lung disease, ICS- inhaled corticosteroids, LABA- long-term bronchodilators, LAMA-long-term tiotropium bromide, SBP-systolic blood pressure, SPO^2^-saturation of peripheral blood oxygen.

^†^*P* < 0.05 vs. controls

^††^*P* < 0.01 vs. controls

*P < 0.05 vs. no cor pulmonale

**P < 0.01 vs. no cor pulmonale.

Among the 81 patients with COPD, 39 (48.1%) were placed in the group with cor pulmonale and the other 42 (52.9%) in the group without cor pulmonale, based on the TAPSE values. Table 2 shows the RV functional and morphology parameters among the three subgroups. In the subgroup of cor pulmonale, there were 7/32 patients in Gold stages III and IV, respectively. In patients without cor pulmonale, there were 22/20 patients in stages III and IV, respectively. When stratified with the GOLD stages, the data showed that TAPSE values were decreased with the increase in the GOLD stage: the mean value of TAPSE was (18.38 ± 3.12) mm in GOLD stage III and (16.37±3.20) mm in GOLD stage IV, respectively. In comparison, the mean values of TAPSE in controls were (28.28±2.56) mm, and the ANOVA test presented a significant difference in TAPSE values among the three groups, with *P*<0.01. In addition, Doppler detected PAH in 32 (39.5%) patients, with the mean SPAP value of 48.24 mmHg.

**Table 2:** Echocardiographic Data in Controls and Two Subgroups of COPD (Cor Pulmonale and No Cor Pulmonale) (Mean±SD).

Variables	Controls (n = 40)	No cor pulmonale (n = 42)	Cor pulmonale (n = 39)	F value	*P* value
Heart rate	74.85±4.65	82.69±7.30^††^	85.05±5.87^††^	30.88	<0.001
LVEF (%)	71.55±5.99	68.29±6.65†	66.76 ±6.90^††^	5.89	0.004
SPAP (mmHg)	23.60±2.32	31.67±5.37^††^	45.10±9.03^††^**	122.41	<0.001
TAPSE (mm)	28.28±2.56	19.67±2.58^††^	14.31±0.66^††^**	425.27	<0.001
FAC (%)	48.00±3.57	41.31±4.91^††^	33.51±2.98^††^**	134.49	<0.001
RVMPI	31.13±3.02	45.74±5.70^††^	55.10±7.57^††^**	163.70	<0.001
RVWT (mm)	3.75±0.81	4.73±0.83^††^	5.77±0.97^††^**	53.26	<0.001
RAD (cm)	35.28±3.30	40.19±5.71^††^	42.95±4.93^††^*	26.32	<0.001
RVD (cm)	20.73±1.47	33.07±3.72^††^	34.77±2.92^††^*	284.11	<0.001
Myostatin(ng/ml)	8.79±2.79	13.56±3.09^††^	16.68 ± 2.95^††^**	72.23	<0.001
BNP (pg/ml)	18.37±7.15	111.00±55.73^††^	142.18±42.55^††^*	98.25	<0.001

BNP- B-type natriuretic peptide, COPD-chronic obstructive pulmonary disease, FAC-fractional area change, LVEF-left ventricular ejection fraction, RAD-right atria diameter, RVD-right ventricular diameter, RVMPI-right ventricular myocardial performance index, RVWT-right ventricular free wall thickness. SPAP-systolic pulmonary artery pressure, TAPSE-tricuspid annular plane systolic excursion.

^†^*P* < 0.05 vs. controls

^††^*P* < 0.01 vs. controls

*P < 0.05 vs. no cor pulmonale

**P < 0.01 vs. no cor pulmonale.

### Plasma Myostatin and BNP Levels

**Fig 1:**
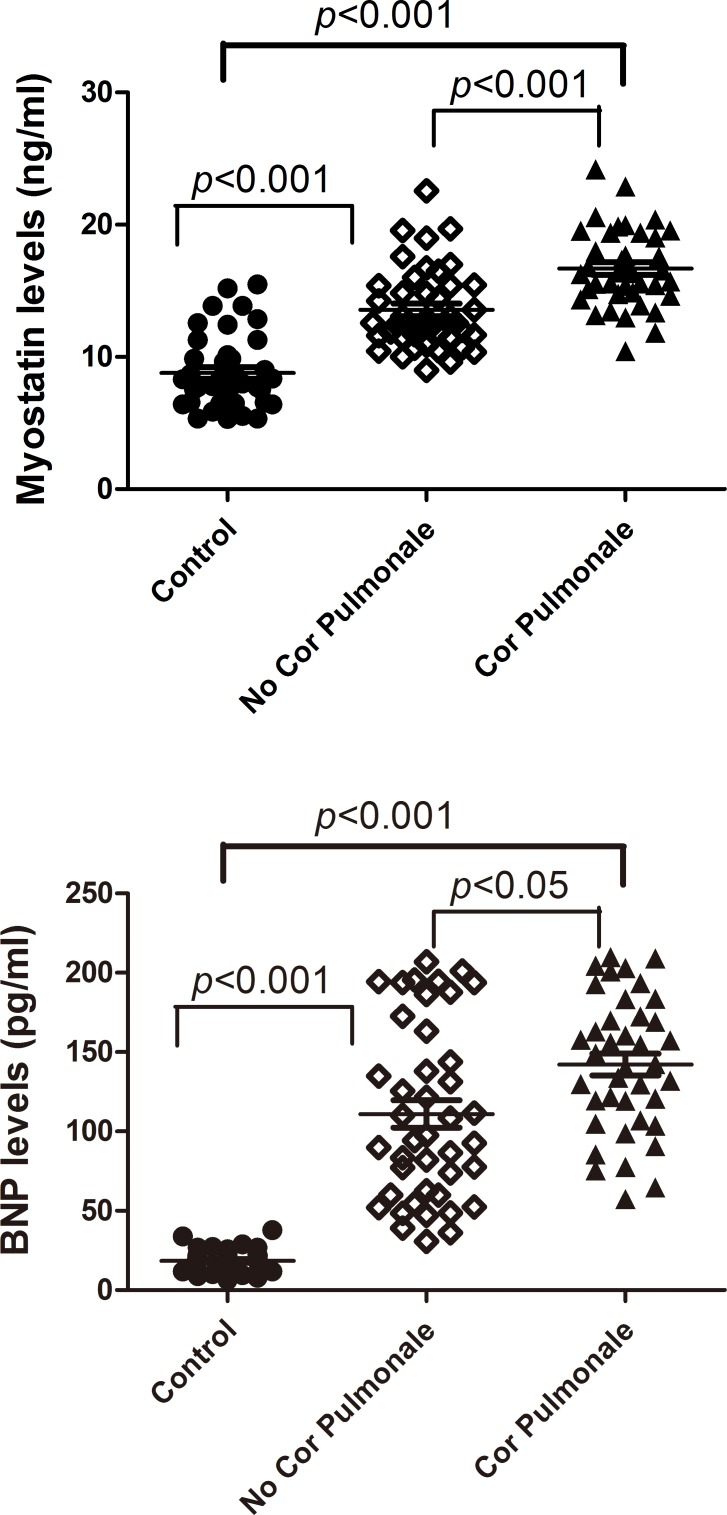
Fig 1(A) and (B). Comparison of myostatin and B-type natriuretic peptide (BNP) levels among the three groups. Circles represent control group, diamonds represent the group without cor pulmonale, and triangles represents the group with cor pulmonale. (A) Significantly higher myostatin levels in group without cor pulmonale, and much higher levels in the group with cor pulmonale, when compared with controls. (B) Significant higher BNP levels in the group without cor pulmonale, and much higher levels in the group with cor pulmonale, when compared with controls.

Plasma myostatin levels were higher in the COPD patients than in controls, especially in those with cor pulmonale (Table 1). Fig 1 shows significant differences in plasma myostatin levels among the three groups including controls, COPD with cor pulmonale, and COPD without cor pulmonale (*P* < 0.001). Significant differences were also noted in plasma BNP levels among the three groups, which is shown in Table 1 and Fig 1B.

### Correlations

Pearson's correlation analysis revealed that plasma myostatin levels were significantly correlated inversely with TAPSE values and FAC, but positively with RVMPI and SPAP among the patients with COPD, with each *P*< 0.01. For the association with RV morphology parameters, plasma myostatin levels were found to be mildly correlated with both RV free wall thickness and RV diameter in the COPD group (each *P* < 0.05). The Pearson's correlation coefficients are shown in Table 3. In addition, there was an inverse correlation between plasma myostatin levels and BMI (*r* = -0.25, *p* = 0.024) in COPD patients. Among these parameters, multivariate stepwise regression analysis suggested that only TAPSE (-0.32) and RVMPI (0.28) were the independent factors that were significantly correlated with plasma myostatin levels, with *R*^2^ of 0.25 (*p*<0.001).

**Table 3:** Correlation Coefficients of Plasma Myostatin and BNP levels with Echocardiographic Parameters in COPD Patients.

	Myostatin		BNP	
	r-value	P-value	r value	P-value
TAPSE	-0.457	<0.001	-0.278	0.012
FAC	-0.435	<0.001	-0.223	0.045
RVMPI	0.442	<0.001	0.193	0.085
PASP	0.394	0.001	0.306	0.005
RVWT	0.331	0.003	0.211	0.059
RAD	0.210	0.072	0.024	0.832
RVD	0.294	0.012	0.084	0.456

BNP- B-type natriuretic peptide, COPD-chronic obstructive pulmonary disease, LVEF-left ventricular ejection fraction, FAC-fractional area change, RAD-right atria diameter, RVD-right ventricular diameter, RVMPI-right ventricular myocardial performance index, RVWT-right ventricular free wall thickness. SPAP-systolic pulmonary artery pressure, TAPSE-tricuspid annular plane systolic excursion.

**Fig 2:**
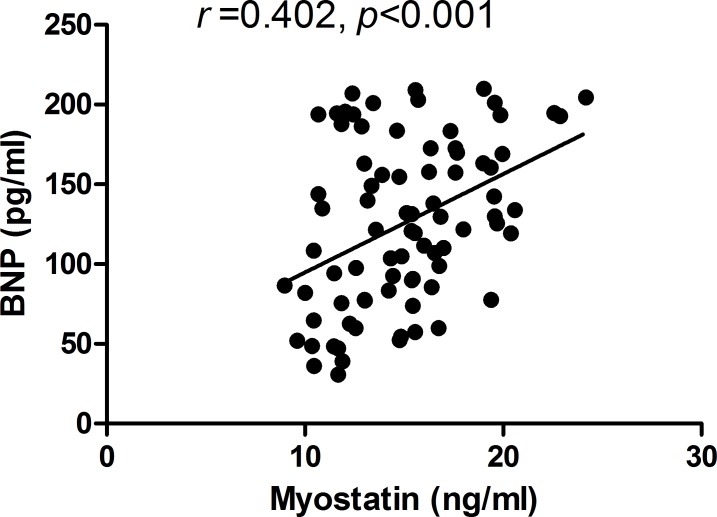
Relationship between plasma myostatin and B-type natriuretic peptide (BNP) levels: a significant correlation was observed between them in patients with COPD.

A significant correlation of plasma BNP levels with myostatin levels was observed (*r* = 0.40, *P* < 0.001) in the COPD group, which is shown in Fig 2. In addition, mild but significant correlations of BNP levels with SPAP, FAC, and TAPSE values were observed among the COPD patients (each *P* < 0.05); however, the correlations were not so much strong when compared with myostatin levels, based on the correlation coefficients (*r*). These correlation coefficients are shown in Table 3. As for RV morphology, none of the parameters was found to be correlated with plasma BNP levels. Multivariate stepwise regression analysis suggested that only SPAP were significantly correlated with plasma BNP levels, with *R*^2^ of 0.08 (*p*<0.001).

## Discussion

The present study revealed three main findings. First, plasma myostatin levels were higher in patients with COPD and much higher in those who had cor pulmonale, when compared with controls. Second, plasma myostatin levels were correlated with functional and morphological parameters of the right heart in advanced COPD. Finally, myostatin levels were strongly correlated with indexes for RV dysfunction and hypertrophy compared with BNP levels in advanced COPD, based on the correlation coefficients. The present study was the first to investigate the association between plasma myostatin levels and echocardiographic parameters of the right heart in patients with stable COPD.

Most of the patients with COPD recruited in this study had very severe airflow limitation, with the mean value of FEV_1_%pred less than 30%. Among all the patients, the number of those who had cor pulmonale was 39 (48.1%), indicating that cor pulmonale is prevalent in patients with advanced COPD. RV dysfunction and resultant decompensation of cor pulmonale are the natural course of COPD and prominent in advanced COPD [25], [26]. Among the subgroup of cor pulmonale, some patients had normal pulmonary pressure (PASP < 37mmHg), according to the echocardiogram measurements. The result was in agreement with the study by Hilde et al [5]; they reported that RV dysfunction, RV hypertrophy, and dilation were present even in COPD patients who did not have PAH. The findings of this study also emphasized that the cardiac complications on the right side could start early in the course of the pulmonary vascular disease. The other consistent studies revealed that subclinical RV dysfunction is really prevalent in patients with COPD [27], [28]. As a matter of fact, several risk factors of COPD such as tobacco smoking can induce RV remodeling early in the course of COPD, which has been demonstrated by animal models [29]. Most of the patients in this study were ex-smokers, so it was not surprising that the number of patients (39) with RV dysfunction was more than the number of those who had PAH (32) in the present study.

One of the key findings in the present study was that myostatin levels increased remarkably in the 39 patients with COPD who had cor pulmonale compared with controls, and the levels were also significantly higher compared with patients who did not have cor pulmonale. This finding was similar to the changes reported in other studies that focused on the left heart failure. Gruson et al [13] reported the elevated plasma myostatin levels in patients with chronic congestive heart failure compared with controls. Also, George et al [12] found that plasma myostatin levels significantly increased in patients with heart failure, although the heart failure was caused by ischemic heart disease or dilated cardiomyopathy. A further finding of this study was that plasma myostatin levels were significantly correlated with important echocardiographic parameters for RV function (TAPSE values and RVMPI) in COPD patients. The results indicated that the elevated levels of circulating myostatin might be associated with RV dysfunction in advanced COPD. Myostatin, a regulator of skeletal muscle mass, is well known to be mainly expressed in skeletal muscles, while it is also expressed in the myocardium. The results of this study raised a question: What was the recourse of the elevated circulation myostatin in the present study? Did heart secrete myostatin in heart failure patients?

Myostatin was reported to be expressed in the myocardium for the first time in 1999, when Sharma et al found that myostatin was upregulated in cardiomyocytes after infarction in animal models [30]. Later, more evidences showed elevated myostatin levels in the myocardium of animals with heart injury and/or heart failure [31], [32], [33]. Moreover, a very recent animal model study has shown not only that myocardium myostatin levels increase in rats with heart failure, but also that intervention therapy can decrease myostatin levels in the myocardium [34]. Since myostatin levels have also been demonstrated to be simultaneously increased in the circulation and myocardium of patients with heart failure [12], this study supposes that the elevation of circulating myostatin might be accompanied by the increase in cardiac myostatin, and the latter was the source of elevated circulating myostatin in patients with RV dysfunction in this study, which is supported by previous studies. Heineke et al [35] reported that cardiac-specific overexpression of myostatin in heart failure could increase circulating levels of myostatin by three- to fourfold, leading to an increase in circulating myostatin levels and a reduction in weight of the peripheral muscles. The report also explained the mildly inverse correlation between the plasma myostatin levels and BMI in patients (*r* = –0.25, *P* = 0.02) in this study. Heineke et al [35] also showed that plasma myostatin levels increased by inducing heart failure in rats with genetic deletion of myostatin in skeletal muscles of rats. Gruson et al [13] demonstrated that the increased myostatin in the myocardium resulted in a spillover of the peptide into the circulation. Moreover, Lenk et al [32] showed that exercise training led to the reduction of the intramuscular myostatin protein, but the serum concentration of myostatin revealed no significant alteration. Combined with these findings, the results of the present study indicate that the increased plasma myostatin could be, at least in part, attributed to the impairment of RV function in the patients.

A previous research showed that the expression of myostatin increased in the myocardium of animal models when heart failure was induced by pressure overload [35]. Consistently, a significantly positive correlation between plasma myostatin levels and SPAP was observed in the patients of this study. In advanced COPD, one of the pathological mechanisms for cor pulmonale is that RV is overloaded with PAH. With the increasing severity of airflow limitation and chronic hypoxemia, pulmonary vascular constriction and resistance rise, and pulmonary arterial pressure increases. This means that RV function is compromised by long-term excessive afterload and mechanical stress, leading to cor pulmonale followed by decompensation of right heart failure [36]. This also explains the significant correlations between myostatin levels and multiple RV functional indexes such as TAPSE, FAC, and RVMPI values among the COPD patients in this study. The associations of plasma myostatin levels with both RV functional parameters and pulmonary hemodynamic index raised the possibility that the expression of myostatin could be upregulated in the right heart of the patients with advanced COPD. In addition, Wang et al [37] reported that the treatment of rat neonatal cardiomyocytes with angiotensin II resulted in an increased expression of myostatin and more activated form of myostatin in the myocardium. Actually, renin–angiotensin system has been recognized to play an important role in developing cor pulmonale [38]. Hence, it is conceivable that the expression of myostatin might be increased in the myocardium of the patients in this study who had RV dysfunction to a varying degree, leading to remarkably higher myostatin levels in the circulation in these patients.

It was expected that an enlarged RV diameter was presented in most of the patients who had cor pulmonale in this study. However, the noticeable finding was that plasma myostatin levels were significantly correlated with RV diameters among the COPD patients, and more significantly correlated with RVMPI, based on the correlation coefficients. Increased RVMPI is an index of RV global dysfunction and also an indirect indicator of RV dilation [39]. The findings of the study result suggested that elevated plasma myostatin levels might be associated with increases in RV volume in advanced COPD. This suggestion could be explained by a study on animal models of heart failure in which the expression of myostatin was raised in the myocardium of rats when heart failure was induced by volume overload [40].

Notwithstanding, the exact role myostatin plays in heart failure is not very clear until now. McKoy et al [41] demonstrated that myostatin can act as an inhibitor of cardiomyocyte hyperplastic growth factors. Consistently, most of the patients in this study with cor pulmonale had a hypertrophied RV with the RV free wall thickness above 5 mm [42]. Moreover, a positive correlation between myostatin levels and the wall thickness was observed in the patients with COPD. As a matter of fact, hypertrophy is the early sign of adaption of RV to the excessive afterload. By thickening the wall, RV tends to normalize the pathological stress. In the course of chronic cor pulmonale, RV initially undergoes hypertrophy, often followed by decompensation of dysfunction, dilatation, and eventual failure. Morissette et al [43] suggested that myostatin may act as a potential counter-regulator in response to pathological stimuli in the activation of heart failure, but this counterbalance is at the expense of ventricular function. So, it is reasonable that the multiple regression correlation analysis suggested RV functional parameters (TAPSE and RVMPI) as the final factors significantly contributing to plasma myostatin levels in the COPD patients in this study. The results suggest that increased plasma myostatin could be, to some extent, an indicator of RV dysfunction in advanced COPD.

Plasma BNP levels, which were analyzed in the present study in comparison with myostatin, were also higher in COPD patients compared with controls, and much higher in those who had cor pulmonale. Pearson correlation analysis also observed significant correlations of BNP levels with SPAP, TAPSE and FAC among the study patients. Elevated plasma BNP levels have been reported in RV dysfunction secondary to chronic respiratory diseases in recent studies [44], [45]. Collectively, the finding of this study emphasized the elevated plasma BNP level to be a useful marker for RV stress. Interestingly, a positive correlation between BNP and myostatin levels was found in the COPD group. This is because both BNP and myostatin are expressed in the myocardium in response to increased wall stress, and both of them can be released from the myocardium into the circulation. Hence, it is reasonable and predictable to have a positive correlation between plasma BNP and myostatin levels. However, correlation coefficients revealed that BNP levels were not as strongly correlated to RV function as myostatin levels; furthermore, regression analysis revealed that two of the important RV functional parameters (TAPSE and RVMPI) correlated significantly with myostatin levels but not with BNP levels. This is worth noticing because BNP is considered as a biomarker of RV dysfunction associated with chronic lung disease [46], [47]. In addition, no significant correlation was found between BNP levels and RV geometry in the present study. Therefore, this study indicates that, in comparison with BNP, myostatin might be a stronger indicator for cor pulmonale in advanced COPD.

## Study Limitations

The present study had several limitations. First, it was supposed that elevated plasma myostatin might come out of the myocardium in COPD patients who had RV dysfunction and/or hypertrophy, but heart biopsy was not performed in the patients. Thus, it was not very clear whether cardiac myostatin levels were elevated in patients with cor pulmonale. Another limitation was that the exact relationship between circulating and myocardial myostatin levels was not investigated. A further study of the RV biopsy in this population would be helpful. However, evidences have already shown that cardiac myostatin levels are increased in dysfunctional left heart in both animals and humans. Moreover, the elevated circulating myostatin has been demonstrated to be released from the myocardium of rats with chronic heart failure. Furthermore, these limitations did not interfere with the finding that plasma levels of myostatin were higher in patients with cor pulmonale and associated with the severity of the RV dysfunction in advanced COPD.

## Conclusion

In summary, the present study has shown that plasma myostatin levels are remarkably increased in COPD patients who had cor pulmonale and correlated with RV function and geometry in advanced COPD. In comparison with plasma BNP, myostatin levels had a stronger correlation with the severity of RV dysfunction. These findings suggest that myostatin might be superior to BNP in the early diagnosis of cor pulmonale in COPD.
